# Circulating inflammatory cytokines and colorectal cancer: New insights from Mendelian randomization

**DOI:** 10.1097/MD.0000000000041331

**Published:** 2025-01-24

**Authors:** Qinglu Fan, Jing Wang, Miao Tian, Abdulla Sawut, Di Xiao, Zuohuizi Yi, Liao Chen

**Affiliations:** a Renmin Hospital of Wuhan University, Wuhan, Hubei Province, People’s Republic of China.

**Keywords:** cancer, colorectal cancer, glial cell line-derived neurotrophic factor, inflammatory cytokines, Mendelian randomization, tumor necrosis factor-related apoptosis-inducing ligand

## Abstract

Colorectal cancer (CRC) is one of the most common cancers worldwide and inflammation is believed to play an important role in CRC. In this study, we comprehensively analyzed the causal association between 91 circulating inflammatory cytokines and the risk of CRC using Mendelian randomization (MR). Based on genome-wide association study summary statistics, we examined the causal effects of 91 circulating inflammatory cytokines on CRC. A series of MR methods, including bidirectional MR, replication sample MR, and multivariable MR, were employed to provide more robust causal estimates. After the validation with 3 MR methods and a series of sensitivity analyses, 2 circulating inflammatory factors were found to be significantly associated with the risk of CRC at the genetic level. Specifically, genetically predicted circulating levels of glial cell line-derived neurotrophic factor (GDNF) (OR = 1.12; 95% CI: 1.05–1.19; *P* = 2.72 × 10^-4^) and tumor necrosis factor-related apoptosis-inducing ligand (TRAIL) (OR = 0.93; 95% CI: 0.91–0.99; *P* = 1.00 × 10^-2^) exerted causal effects on CRC risk. In conclusion, this study suggests that increased circulating levels of GDNF and TRAIL are associated with a higher and lower risk of CRC, respectively. GDNF and TRAIL may be 2 potential therapeutic targets that deserve future investigation.

## 1. Introduction

Colorectal cancer (CRC) is currently the third most common cancer after breast cancer and lung cancer and is also the second leading cause of cancer-related mortality globally.^[1]^ In 2020 alone, there were more than 1.9 million newly diagnosed CRC patients and 930,000 deaths worldwide, and the situation is expected to worsen over the next 20 years.^[2]^ Among all newly diagnosed CRC patients, 20% exhibit metastasis at the time of diagnosis, and an additional 25% initially presenting localized disease will develop metastases, with a dismal 5-year survival rate of <20%.^[3]^ Despite some progress in CRC treatment over recent years, prevention and early intervention still play an irreplaceable role in reducing CRC deaths.

Recently, increasing evidence suggests that circulating inflammatory cytokines might be related to the pathogenesis of CRC.^[4]^ For example, studies have shown that CRC patients had significantly higher circulating interleukin-1 beta (IL-1B) levels than healthy controls, and individuals with more advanced stages of CRC also tended to have higher IL-1B levels than those in their early stages.^[5–7]^ Besides, a recent study reviewed the current evidence and summarized that IL-1B could increase VEGF expression and angiogenesis in CRC and could promote tumor growth and invasion through Wnt, Zeb1, and COX2.^[8]^ Other studies reported that higher circulating interleukin-6 (IL-6) levels were related to a higher risk of CRC and elevated circulating IL-6 levels were associated with a poor prognosis for patients with CRC.^[9,10]^ In addition, a recent study has demonstrated that IL-6 regulates autophagy through the IL-6/JAK2/BECN1 pathway and promotes chemotherapy resistance in CRC.^[11]^ However, studies have also yielded inconsistent results on the relationship between certain cytokines and CRC risk. As another example, a study observed a higher level of tumor necrosis factor-alpha (TNF-alpha) in CRC patients, compared with healthy controls, while another study found no difference.^[8,12]^ Given the ongoing debates regarding these circulating inflammatory cytokines and the inherent limitations in traditional observational studies,^[13]^ it remained imperative to evaluate the potential causal relationships between circulating inflammatory cytokines with CRC.

Mendelian randomization (MR) is a novel epidemiological analysis method developed in recent years. By utilizing the summary data of genome-wide association studies (GWAS), MR analysis converts genetic variants as instrumental variables (IVs) for exposures (e.g., a risk factor) and explores their potential causal relationships with specific outcomes.^[14]^ Since IVs are randomly allocated at meiosis and are usually not influenced by confounding factors and reverse causation, MR analysis can be used to make causal inference in a reliable manner.^[15]^ Recently, a GWAS summary data encompassing 91 circulating inflammatory cytokines was published openly, which provided an opportunity to investigate their potential associations with CRC.^[16]^

In this study, we conducted a series of MR analyses to comprehensively evaluate the causal relationships between 91 circulating inflammatory cytokines and CRC risk, hopefully to provide some new insights into the pathogenesis and early prevention of CRC.

## 2. Methods

### 2.1. Study design

**Figure 1.**
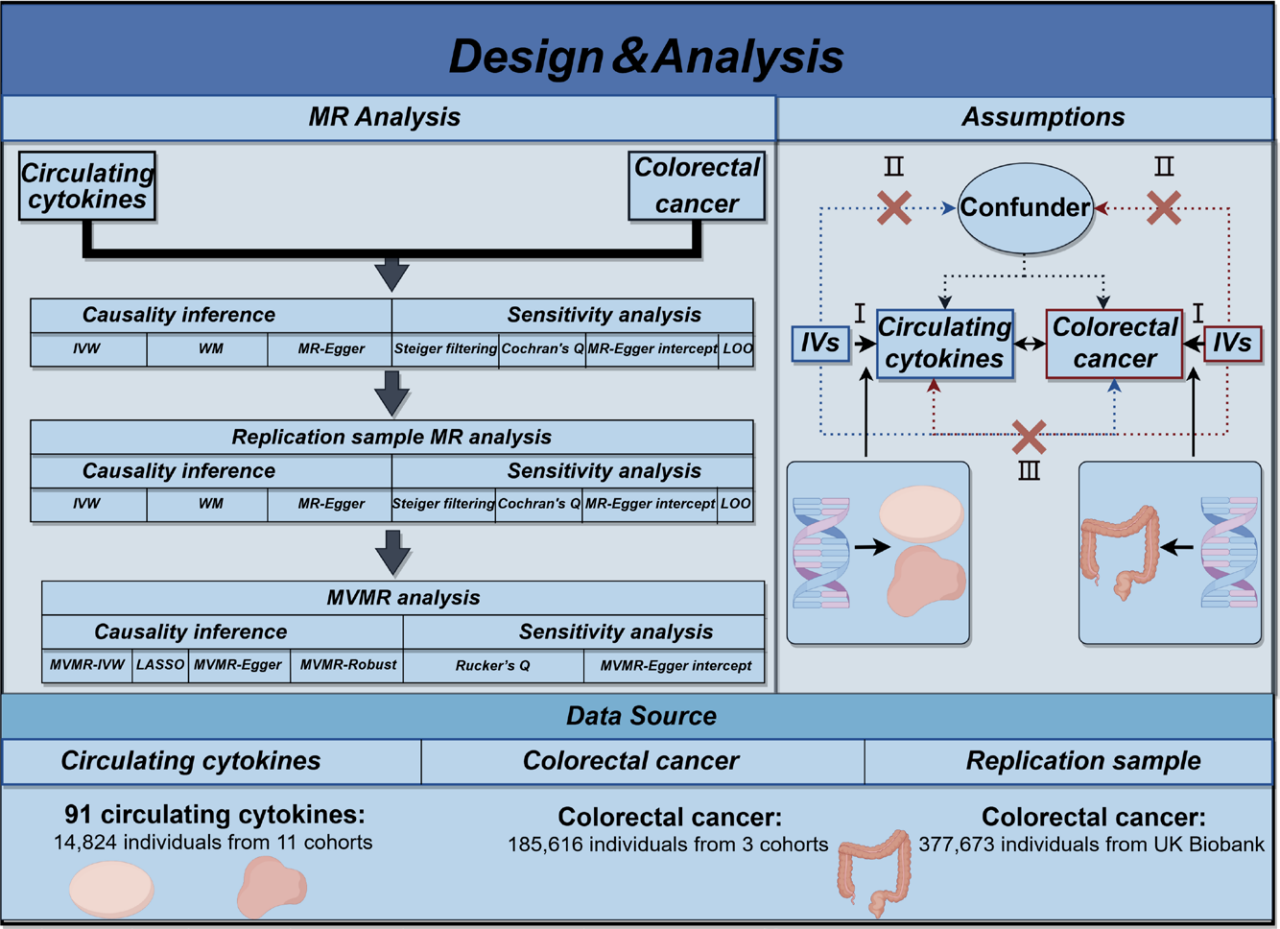
An overview of the study design. IVs = instrumental variables; IVW = inverse variance weighted; LASSO = least absolute shrinkage and selection operator; LOO = leave-one-out; MR = Mendelian randomization; MVMR = multivariable Mendelian randomization; WM = weighted median.

No ethical review was needed due to the GWAS data were publicly accessed and no new data were collected. The flow chart of this MR analysis study is displayed in Figure 1. First of all, a bidirectional MR analysis was conducted to infer the causality between each circulating inflammatory cytokines levels with CRC. Then, a replication sample MR was performed to further verify the robustness of the causal relationships, which survived the bidirectional MR analysis. Finally, a multivariable MR (MVMR) analysis was utilized to estimate the independent effect of the causal relationships meeting the above MR analysis on CRC. At the same time, MR research relies on 3 fundamental premises: (i) IVs are associated with the exposure; (ii) IVs are independent of confounders; (iii) exposure is the only factor that mediates IVs–outcome associations.^[17]^

### 2.2. Data Source

Summary data for circulating inflammatory cytokines levels were obtained from the last published GWAS dataset with up to 14,824 participants of European descent.^[16]^ Detailed information about this GWAS data is shown in Table S1, Supplemental Digital Content, http://links.lww.com/MD/O294. Summary data for CRC GWAS were extracted from the published dataset, which included a total of 185,616 European participants.^[18]^ In the replication phase, summary data for CRC replication sample was retrieved from the IEU OPEN GWAS project (https://gwas.mrcieu.ac.uk/; GWAS ID ieu-b-4965)^[19]^ with 377,673 participants of European ancestry.

### 2.3. Selection of IVs

Single nucleotide polymorphisms (SNPs) meeting the following criteria were selected as IVs: (1) SNPs without linkage disequilibrium (threshold *r*^2^ = 0.001, KB = 10000) and with significance threshold 5 × 10^-8^ and 5 × 10^-6^ were selected for CRC and circulating inflammatory cytokines, respectively.^[20]^ (2) SNPs with the F statistic ≥ 10 (F = β_exposure_^2/SE_exposure_^2) were included, indicating evidence of strong instrumentation, and all palindromic SNPs were dropped;^[21]^ (3) PhenoScannerV2, a tool to exclude any IVs associated with confounders, was used to make sure that the included SNPs meet the second assumption of MR. (4) the MR pleiotropy residual sum and outlier (MR-PRESSO) test was performed to estimate potential horizontal pleiotropy and to eliminate the pleiotropy by removing outlier SNPs.^[22]^

### 2.4. MR analysis

In the bidirectional MR and replication sample MR, we performed 3 methods, including random effects/fixed effects inverse variance weighted (RE/FE-IVW), weighted median (WM), and MR-Egger regression to infer the causal effect. The IVW method is calculated by regressing the coefficient from an outcome regression on the IV on that from an exposure regression on the variant and weighting each estimate by the inverse variance of the association between the instrument and the outcome.^[23]^ The WM method can provide consistent estimates when at least 50% of the weighted variances are from valid IVs.^[24]^ The MR-Egger regression method allows pleiotropy to be present in more than 50% of IVs with less precision.^[25]^ Since the IVW method was usually more efficient than the WM and MR-Egger regression method, the IVW method was used as the main method, supplemented by the WM method and MR-Egger regression method.^[24,26,27]^ Then, to exam the robustness of the causal effect, we performed several sensitivity analyses, including Steiger filtering, the Cochran *Q* test, the MR-Egger intercept test, as well as the leave-one-out (LOO) analysis. The Steiger filtering was conducted to ensure the directionality of the causal association between exposures and outcomes.^[28]^ The Cochran *Q* test was performed to estimate the heterogeneity among IVs associated with each phenotype. The RE-IVW method was used when *P* < .05, and the FE-IVW method was used when *P* > .05.^[29]^ The MR-Egger intercept test was performed to detect the potential horizontal pleiotropy.^[25]^ The LOO analysis was conducted to determine whether the significant results were influenced by any single SNP.^[23]^

In the MVMR analysis, the MVMR-IVW method was utilized as the main analysis,^[30]^ supplemented by the least absolute shrinkage and selection operator regression, MVMR-Egger and MR-Robust method.^[31,32]^ For sensitivity analyses, the Rucker *Q* test was utilized to estimate the heterogeneity,^[33]^ where *P* < .05 suggested using MVMR-RE-IVW method. For the potential pleiotropy, the MVMR-Egger method was used.^[34]^

### 2.5. Statistics analysis

All MR analyses were conducted in R statistical software (version 4.3.0) using the “TwoSampleMR” package (version 0.5.6), “MendelianRandomization” package (version 0.7.0), and “MRPRESSO” package (version 1.0). The results of MR analysis were expressed as odd ratio (OR) with 95% CI to quantify the causal effect of circulating inflammatory cytokines levels, as the exposure, on CRC as the outcome. In the results of reverse MR analysis with CRC as the exposure and circulating inflammatory cytokines levels as the outcome, the results were expressed as β with 95% CI. Bonferroni correction was used for multiple testing correction with *P* < 5.49 × 10^-4^ (0.05/91) as the significant level, and *P* ≥ 5.49 × 10^-4^ and < .05 were considered suggestively significant in the forward MR analysis.^[35]^ In replication sample MR analysis and MVMR analysis, *P* value < .05 was defined as the significant level.^[36]^

## 3. Results

### 3.1. Bidirectional MR analysis

#### 3.1.1. Forward MR analysis from circulating inflammatory cytokines levels to CRC risk

In the forward MR analysis, 1239 IVs met the screening criteria, with the F-statistics ranging from 20.84 to 3549.83, presenting strong association strength. The information of the included IVs is listed in Table S2, Supplemental Digital Content, http://links.lww.com/MD/O294.

**Figure 2.**
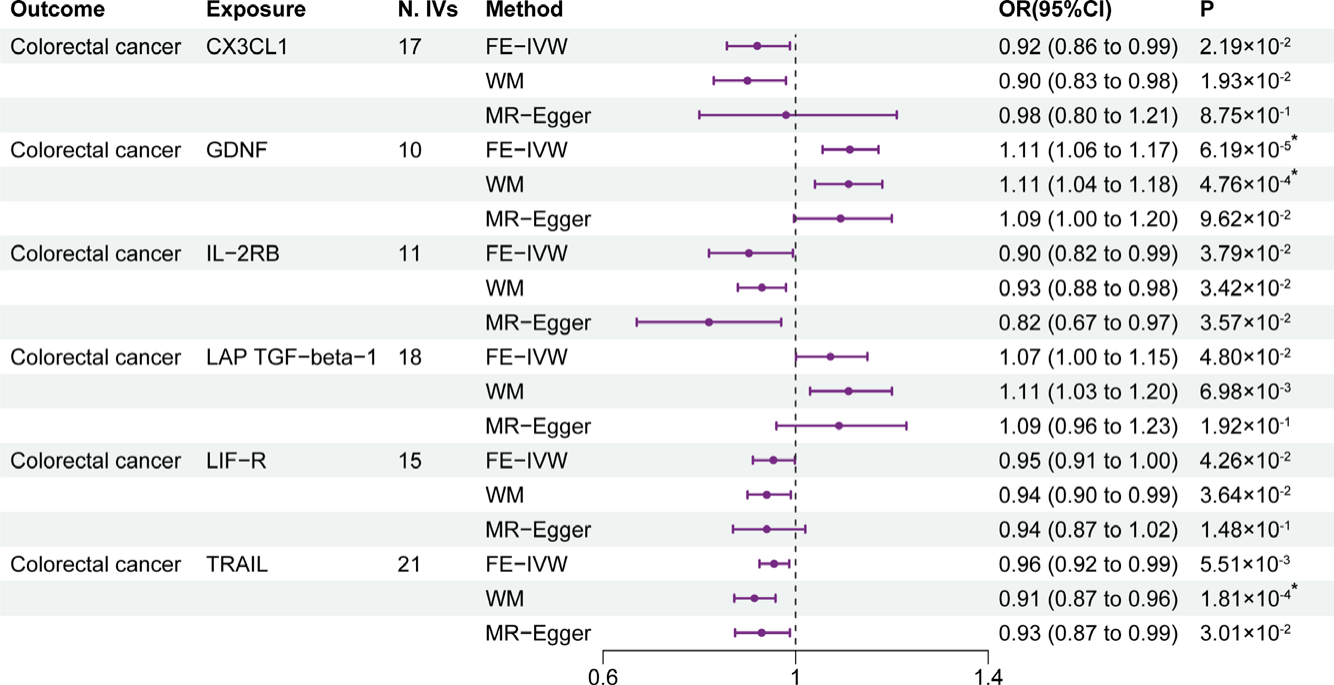
Results of the forward MR analysis. CX3CL1 = C-X3-C motif chemokine ligand 1; FE-IVW = fixed effects inverse variance weighted; GDNF = glial cell line-derived neurotrophic factor; IL-2RB = interleukin-2 receptor subunit beta; LAP TGF-beta-1 = latency-associated peptide transforming growth factor beta 1; LIF-R = leukemia inhibitory factor receptor; N. IVs = the number of IVs; TRAIL = tumor necrosis factor-related apoptosis-inducing ligand; WM = weighted median. *P of statistical significance (<5.49 × 10^-4^).

Results of the forward MR analysis from circulating inflammatory cytokines levels to CRC risk are listed in Figure 2, with a total of 6 circulating inflammatory cytokines identified as possible causal exposures to CRC. Specifically, the IVW method for genetic prediction revealed that increased circulating levels of C-X3-C motif chemokine ligand 1 (CX3CL1) (OR = 0.92; 95% CI: 0.86–0.99; *P* = 2.19 × 10^-2^), interleukin-2 receptor subunit beta (IL-2RB) (OR = 0.90; 95% CI: 0.82–0.99; *P* = 3.79 × 10^-2^), leukemia inhibitory factor receptor (LIF-R) (OR = 0.95; 95% CI: 0.91–1.00; *P* = 4.26 × 10^-2^), and TNF-related apoptosis-inducing ligand (TRAIL) (OR = 0.96; 95% CI: 0.92–0.99; *P* = 5.51 × 10^-3^) were associated with a decreased risk of CRC. However, genetically predicted 1-SD increase in glial cell line-derived neurotrophic factor (GDNF) (OR = 1.11; 95% CI: 1.06–1.17; *P* = 6.19 × 10^-5^), and latency-associated peptide transforming growth factor beta 1 (LAP TGF-beta-1) (OR = 1.07; 95% CI: 1.00–1.15; *P* = 4.80 × 10^-2^) were related to a higher CRC risk. The Steiger filtering did not show any causal effect of CRC on the levels of circulating inflammatory cytokines (Table S3, Supplemental Digital Content, http://links.lww.com/MD/O294). The results from the Cochran *Q* test are shown in Table S3, Supplemental Digital Content, http://links.lww.com/MD/O294 and no heterogeneity was observed. Moreover, no pleiotropy was observed in the MR-Egger intercepts test (Table S3, Supplemental Digital Content, http://links.lww.com/MD/O294). Additionally, no IVs significantly influencing the overall outcome were identify by the LOO analysis (Figure S1, Supplemental Digital Content, http://links.lww.com/MD/O293). The Bonferroni correction showed that higher circulating levels of GDNF (IVW; OR = 1.11; 95% CI: 1.06–1.17; *P* = 6.19 × 10^-5^) and TRAIL (WM; OR = 0.91; 95% CI: 0.87–0.96; *P* = 1.81 × 10^-4^) stayed significantly related to an increased and decreased CRC risk, respectively.

#### 3.1.2. Reverse MR analysis from CRC to identified circulating inflammatory cytokines levels

In the reverse MR analysis, 636 SNPs were selected as IVs for CRC to identified circulating inflammatory cytokines levels, with F-statistics ranging from 29.90 to 516.42. Detailed information for the selected IVs can be found in Table S4, Supplemental Digital Content, http://links.lww.com/MD/O294.

**Figure 3.**
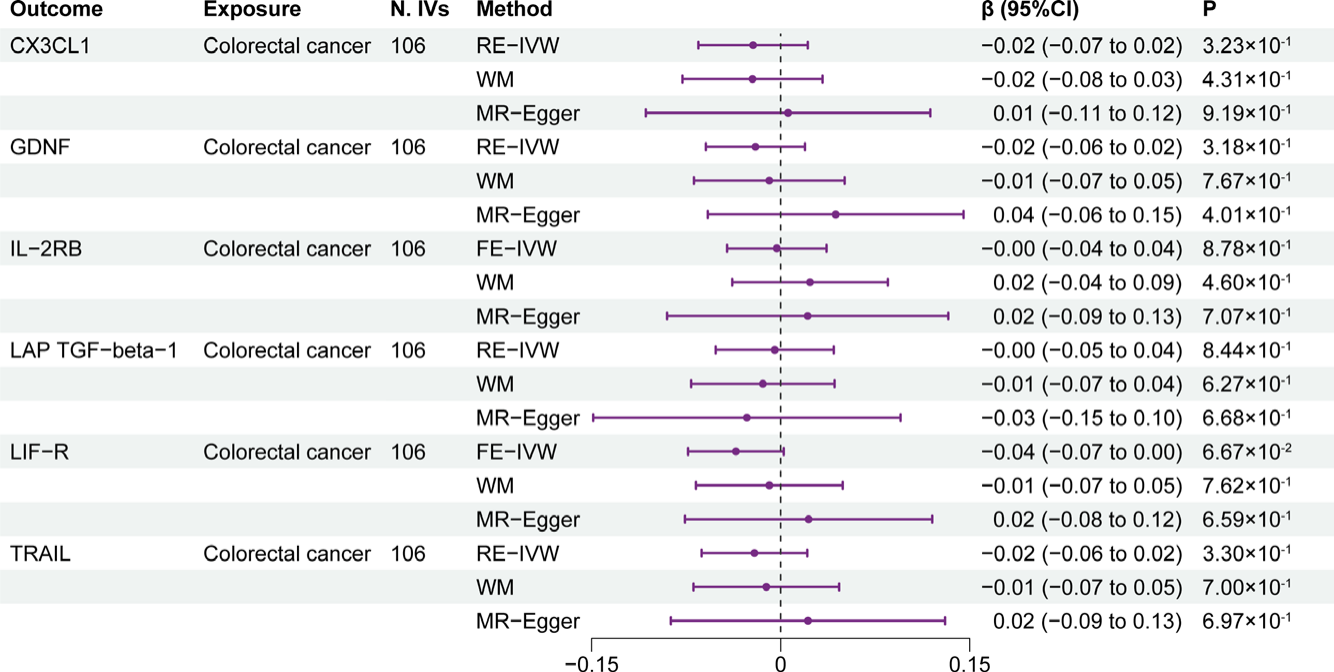
Results of the reverse MR analysis. CX3CL1 = C-X3-C motif chemokine ligand 1; FE-IVW = fixed effects inverse variance weighted; GDNF = glial cell line-derived neurotrophic factor; IL-2RB = interleukin-2 receptor subunit beta; LAP TGF-beta-1 = latency-associated peptide transforming growth factor beta 1; LIF-R = leukemia inhibitory factor receptor; N. IVs = the number of IVs; RE-IVW = random effects inverse variance weighted; TRAIL = tumor necrosis factor-related apoptosis-inducing ligand; WM = weighted median.

The reverse MR analysis results are listed in Figure 3. No association was observed between CRC and the 6 identified circulating inflammatory cytokines levels. Specifically, the IVW method for genetic prediction revealed that CRC were not associated with CX3CL1 (β = −0.02; 95% CI: −0.07 to 0.02; *P* = 3.23 × 10^-1^), GDNF (β = -0.02; 95% CI: −0.06 to 0.02; *P* = 3.18 × 10^-1^), IL-2RB (β = 0.00; 95% CI: −0.04 to 0.04; *P* = 8.78 × 10^-1^), LAP TGF-beta-1 (β = 0.00; 95% CI: −0.05 to 0.04; *P* = 8.44 × 10^-1^), LIF-R (β = -0.04; 95% CI: −0.07 to 0.00; *P* = 6.67 × 10^-2^), and TRAIL (β = -0.02; 95% CI: −0.06 to 0.02; *P* = 3.30 × 10^-1^). The results from the Cochran *Q* test are shown in Table S5, Supplemental Digital Content, http://links.lww.com/MD/O294, with heterogeneity in the analyses of CX3CL1, GDNF, LAP TGF-beta-1, and TRAIL, hence, the RE-IVW method was utilized. Additionally, no evidence of horizontal pleiotropy was observed (Table S5, Supplemental Digital Content, http://links.lww.com/MD/O294).

### 3.2. Replication sample MR analysis

In the replication sample MR analysis from identified circulating inflammatory cytokines levels to CRC, 99 IVs meeting the screening criteria were included, with the F-statistics ranging from 20.86 to 475.40. The information of the included IVs is listed in Table S6, Supplemental Digital Content, http://links.lww.com/MD/O294.

**Figure 4.**
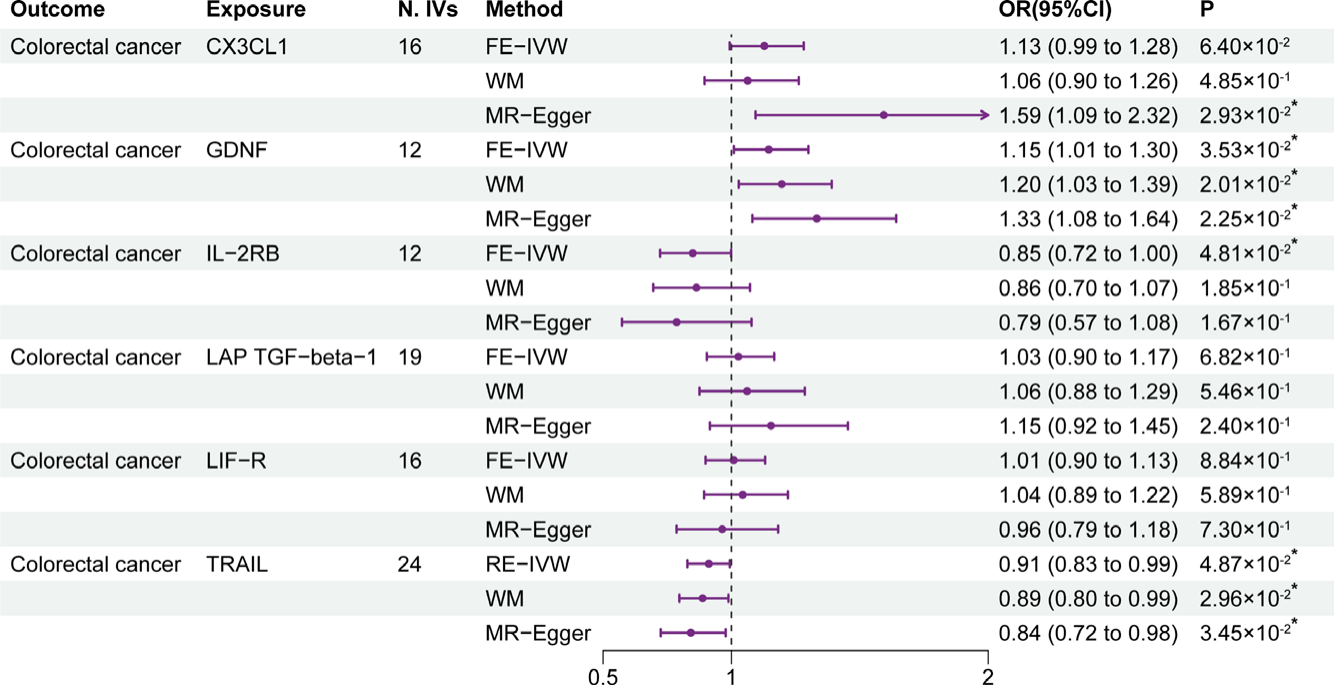
Results of the replication sample MR analysis. CX3CL1 = C-X3-C motif chemokine ligand 1; FE-IVW = fixed effects inverse variance weighted; GDNF = glial cell line-derived neurotrophic factor; IL-2RB = interleukin-2 receptor subunit beta; LAP TGF-beta-1 = latency-associated peptide transforming growth factor beta 1; LIF-R = leukemia inhibitory factor receptor; N. IVs = the number of IVs; RE-IVW = random effects inverse variance weighted; TRAIL = tumor necrosis factor-related apoptosis-inducing ligand; WM = weighted median. *P of statistical significance (<.05).

Results of the replication MR analysis are shown in Figure 4, with the results of 3 identified circulating inflammatory cytokines successfully replicated. Specifically, GDNF (OR = 1.15; 95% CI: 1.01–1.30; *P* = 3.53 × 10^-2^), IL-2RB (OR = 0.85; 95% CI: 0.72–1.00; *P* = 4.81 × 10^-2^), and TRAIL (OR = 0.91; 95% CI: 0.83–0.99; *P* = 4.87 × 10^-2^) were successfully validated in IVW method. However, CX3CL1 (OR = 1.13; 95% *CI*: 0.99–1.28; *P* = 6.40 × 10^-2^), LAP TGF-beta-1 (OR = 1.03; 95% CI: 0.90–1.17; *P* = 6.82 × 10^-2^), and LIF-R (OR = 1.01; 95% CI: 0.90–1.13; *P* = 8.84 × 10^-2^) were not successfully validated. Besides, the Steiger filtering passed the causal direction from identified circulating inflammatory cytokines to CRC (Table S7, Supplemental Digital Content, http://links.lww.com/MD/O294). The results of the Cochran *Q* test are shown in Table S7, Supplemental Digital Content, http://links.lww.com/MD/O294, and heterogeneity was observed in the TRAIL analysis, hence, the RE-IVW method was used. No pleiotropy was observed in the MR-Egger intercepts test (Table S7, Supplemental Digital Content, http://links.lww.com/MD/O294). Additionally, the LOO analysis is shown in Figure S2, Supplemental Digital Content, http://links.lww.com/MD/O293.

### 3.3. MVMR analysis

**Figure 5.**
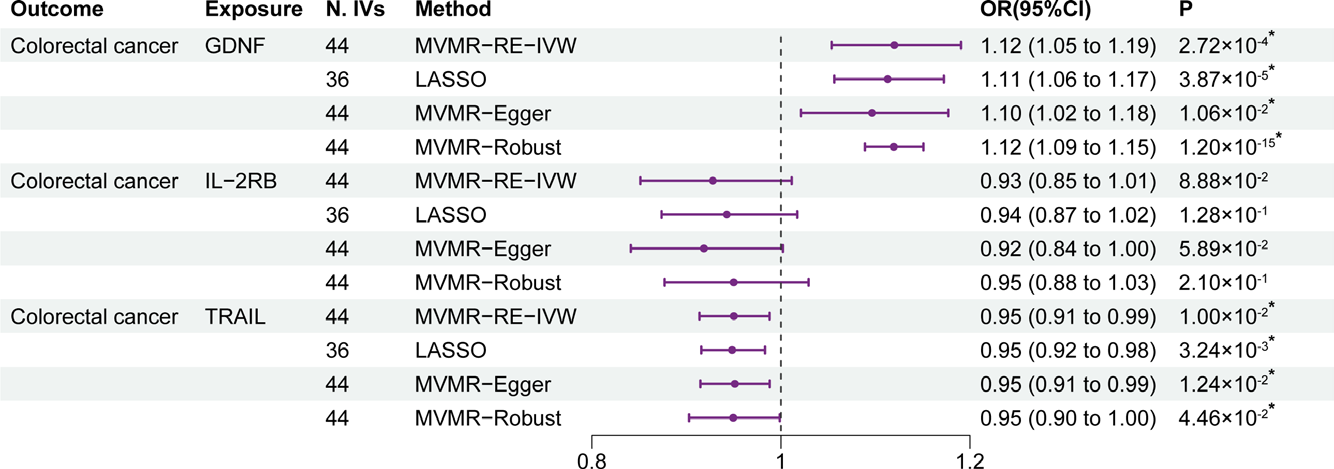
Results of the MVMR analysis. GDNF = glial cell line-derived neurotrophic factor; IL-2RB = interleukin-2 receptor subunit beta; LASOO = least absolute shrinkage and selection operator; MVMR-RE-IVW = multivariable Mendelian randomization fixed effects inverse variance weighted; N. IVs = the number of IVs; TRAIL = tumor necrosis factor-related apoptosis-inducing ligand. *P of statistical significance (<.05).

To estimate the independent causal effects of GDNF, IL-2RB, and TRAIL on CRC, an MVMR analysis was conducted. In the MVMR analysis, 44 SNPs were included as IVs (Table S8, Supplemental Digital Content, http://links.lww.com/MD/O294). The MVMR analysis results are listed in Figure 5, with GDNF and TRAIL staying genetically related to CRC, which suggested that GDNF and TRAIL independently exerted a causal effect on CRC risk. Specifically, circulating GDNF levels (OR = 1.12; 95% CI: 1.05–1.19; *P* = 2.72 × 10^-4^) remained causally positively associated with CRC risk. However, TRAIL (OR = 0.95; 95% CI: 0.91–0.99; *P* = 1.00 × 10^-2^) remained causally related to a decreased risk of CRC. However, the causal effect of IL-2RB on CRC was no longer statistically significant (OR = 0.93; 95% CI: 0.85–1.01; *P* = 8.88 × 10^-2^). In addition, in the Rucker *Q* test (Table S9, Supplemental Digital Content, http://links.lww.com/MD/O294), heterogeneity was observed, as a result of which the MVMR-RE-IVW method was used. Besides, no pleiotropy was observed in the MVMR-Egger intercepts test (Table S9, Supplemental Digital Content, http://links.lww.com/MD/O294).

## 4. Discussion

In the study, we conducted a series of MR analyses to explore the potential causal effect of 91 circulating inflammatory cytokines levels on the risk of CRC. To the best of our knowledge, this study is currently the most comprehensive analysis on the association between circulating inflammatory cytokines and CRC to date.

Firstly, using a bidirectional MR analysis, we found that the genetically predicted circulating levels of CX3CL1, GDNF, IL-2RB, LAP TGF-beta-1, LIF-R, and TRAIL were associated with CRC risk. Among them, GDNF (IVW; OR = 1.11; 95% CI: 1.06–1.17; *P* = 6.19 × 10^-5^) and TRAIL (WM; OR = 0.91; 95% CI: 0.87–0.96; *P* = 1.81 × 10^-4^) passed the Bonferroni correction. Then, we further performed a replication sample MR analysis, which successfully verified the causality between GDNF (IVW; OR = 1.15; 95% CI: 1.01–1.30; *P* = 3.53 × 10^-2^), IL-2RB (IVW; OR = 0.85; 95% CI: 0.72–1.00; *P* = 4.81 × 10^-2^), and TRAIL (IVW; OR = 0.91; 95% CI: 0.83–0.99; *P* = 4.87 × 10^-2^) with CRC. Lastly, considering the potential interrelationships between GDNF, IL-2RB, and TRAIL, an MVMR analysis was performed to evaluate whether the above causal effects were independent of each other. Results of MVMR showed that the causal effect of GDNF (IVW; OR = 1.12; 95% CI: 1.05–1.19; *P* = 2.72 × 10^-4^) and TRAIL (IVW; OR = 0.93; 95% CI: 0.91–0.99; *P* = 1.00 × 10^-2^) on CRC risk remained statistically significant. After considering the results of the bidirectional MR, replication sample MR, and MVMR analyses, it can be concluded that, compared to individuals with normal circulating inflammatory cytokines levels, these with higher GDNF levels are more likely to suffer from CRC, while higher TRAIL levels will protect individuals from CRC.

GDNF, a 134 amino acid protein, is one of the important components of the neurotrophic networks of the central nervous system.^[37,38]^ Previous investigations have demonstrated the tumor-promoting effect of GDNF on several cancers, such as glioma, gastric cancer, and pancreatic cancer.^[39–41]^ Besides, increased GDNF has also been found in the inflamed gut tissues in patients with inflammatory bowel disease, a well-established precancerous condition for CRC.^[42]^ Another worth notice phenomenon is that that methylation frequency for GDNF gene is higher in CRC tissue samples than normal ones.^[43,44]^ However, more research is needed to determine the precise underlying mechanisms of GDNF in the development of CRC.

TRAIL is a member of the TNF superfamily that can regulate cell apoptosis and entosis through binding to receptors TRAIL-R1/DR4 and TRAIL-R2/DR5.^[45–48]^ So far, TRAIL has a wide range of investigations in oncology and shows significant potential as a novel target for biological anticancer drugs.^[49]^ In our study, we observed that a genetically predicted higher circulating TRAIL level was related to a decreased CRC risk, which is supported by previous studies. For example, Bozkurt et al found that TRAIL can initiate entosis in colon cancer to eliminate cancer cells.^[50]^ TRAIL is also known to induce apoptosis of cancer cells in CRC.^[51,52]^ Besides, it has been found that platelets can promote CRC via PSGL-1/JNK/STAT1 signaling pathway.^[53]^ However, TRAIL can promote platelet apoptosis to inhibit the cell invasion of CRC.^[54]^

To summarize, our results indicate that GDNF and TRAIL exhibit distinct yet promising potentials as therapeutic targets in CRC, each with its unique role. Specifically, GDNF seems to elevate CRC risk, making it a candidate for therapeutic inhibition in patients with CRC. Developing targeted therapies aimed at decreasing GDNF expression or disrupting its signaling pathways could potentially mitigate CRC progression. In addition, circulating GDNF may also serve as a potential biomarker for the early diagnosis of CRC. By contrast, TRAIL demonstrates potential as a protective agent against CRC. With TRAIL-based therapies already being explored in many other cancer types, our findings further justify the investigation of these therapies specifically tailored for CRC.^[49,55,56]^ We recognize that while our findings are promising, further preclinical and clinical studies are still needed.

Nevertheless, several limitations should be noted. First, due to the fact that the included participants are predominantly of European ancestry, caution should be taken when trying to generalize the conclusion to individuals of other races and a further MR analysis focusing on diverse ethnic groups are needed in future exploration. Second, although the MR-PRESSO test was performed to find potential pleiotropy, the possibility of pleiotropy cannot be totally ruled out. Third, the incidence of CRC has obvious gender differences. However, GWAS did not provide sex-stratified data on circulating inflammatory cytokines, and we were therefore unable to study whether circulating inflammatory cytokines have exerted a sex-related effect on CRC, which deserves to be further explored in future research. Fourth, while the causal relationships between circulating inflammatory cytokines with CRC were identified, the underlying mechanism is elusive and further research is necessary. Last but not last least, although we have comprehensively assessed the association between 91 circulating inflammatory proteins and the risk of CRC, there are still some inflammatory proteins that were not included in the analysis. Future research is anticipated to investigate whether these excluded inflammatory proteins may also influence the risk of CRC.

## 5. Conclusion

To conclude, this study revealed that circulating GDNF level was genetically positively related to an increased CRC risk, while higher circulating TRAIL level decreased the risk of CRC. These findings provide promising genetic evidence for potential therapeutic targets in patients with CRC. Additionally, we emphasize the importance of future research to further investigate the underlying mechanisms of these associations, finally aiming to improve the clinical outcomes for CRC patients.

## Acknowledgments

We would like to thank the Editors and Reviewers for their suggestions on improving this manuscript.

## Author contributions

**Conceptualization:** Qinglu Fan, Jing Wang, Miao Tian.

**Data curation:** Qinglu Fan, Jing Wang, Miao Tian.

**Methodology:** Qinglu Fan, Zuohuizi Yi, Liao Chen.

**Supervision:** Liao Chen.

**Visualization:** Qinglu Fan, Di Xiao, Zuohuizi Yi.

**Writing – original draft:** Qinglu Fan, Jing Wang, Miao Tian.

**Writing – review & editing:** Abdulla Sawut, Di Xiao, Zuohuizi Yi, Liao Chen.

## Supplementary Material


