# Supplementary figures and images for: Circulating inflammatory cytokines and colorectal cancer: New insights from Mendelian randomization
Source: Medicine (Baltimore). 2025 Jan 24;104(4):e41331. doi: 10.1097/MD.0000000000041331 (PMC11771603; doi:10.1097/MD.0000000000041331)

Supplementary Figure S1

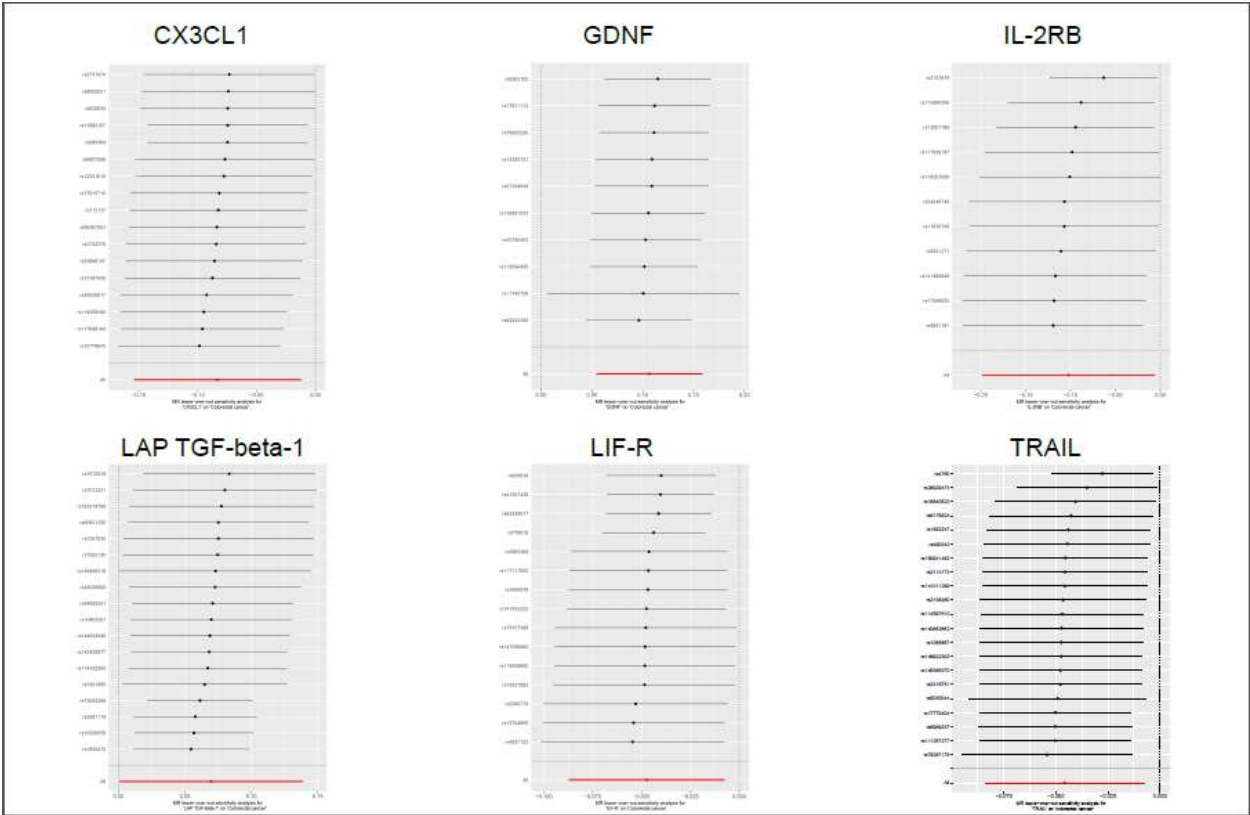

Supplementary Figure S2

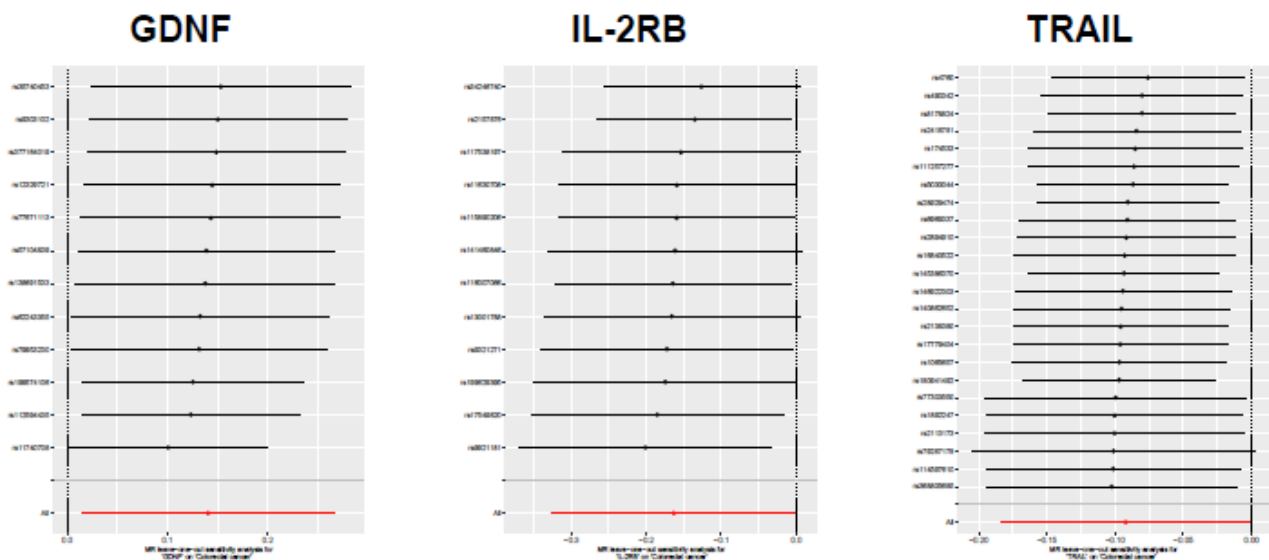

Supplement: Supplementary file 2 [file medi-104-e41331-s002.pdf]
